# Tenosynovial chondromatosis of the flexor pollicis longus tendon: A subtype of primary synovial chondromatosis

**DOI:** 10.1016/j.radcr.2020.12.021

**Published:** 2020-12-18

**Authors:** Alexandra Murphy, Bryan Yelverton, Danilo Vukanic, Zornitsa Tsvetanova, Sarah-Kate Eustace, Alan Molloy, Conor J O'Keane, Eoin Kavanagh

**Affiliations:** a Radiology Department, Cappagh National Orthopaedic Hospital, Cappagh Road, Cappoge, Dublin 11, D11 EV29, Ireland; b Radiology Department, Level 2, Whitty Building, Mater University Hospital, Eccles St, Dublin 7, Do7 R2WY, Ireland; c Orthopaedic Surgery Department, Cappagh National Orthopaedic Hospital, Cappagh Road, Cappoge, Dublin 11, D11 EV29, Ireland; d Histopathology Department, Mater University Hospital, Eccles St, Dublin 7, Do7 R2WY, Ireland; e Histopathology Department, Cappagh National Orthopaedic Hospital, Cappagh Road, Cappoge, Dublin 11, D11 EV29, Ireland

**Keywords:** Tenosynovial chondromatosis, Thumb, Tendon sheath synovial chondromatosis, Flexor pollicis longus tendon, Synovectomy

## Abstract

Primary synovial chondromatosis is a rare benign neoplastic process, in which cartilaginous nodules are produced in the subsynovial tissue. It has 3 main subtypes (intra-articular, tenosynovial and bursal). We present the case of a 61-year-old female, with a mass involving her right thumb for at least 5 years, which had recently increased in size. X-ray showed a soft tissue mass, without calcification or any underlying bony abnormality. Ultrasound and MRI showed a 6-cm mass surrounding the right flexor pollicis longus tendon of the right thumb. The patient went on to have surgical resection and was given a diagnosis of tenosynovial chondromatosis.

## Background/Introduction

Primary synovial chondromatosis is a rare benign neoplastic process, in which cartilaginous nodules are produced in subsynovial tissue [1,2]. It can be intra-articular (within a joint), or less commonly extra-articular; within a tendon sheath (tenosynovial chondromatosis), or bursa (bursal chondromatosis). In intra-articular primary synovial chondromatosis, large joints are generally affected such as the knee, hip, elbow, shoulder and ankle [1,2]. The cartilaginous nodules grow and detach from the subsynovium [1]. They may enlarge over time and if they become calcified, the condition is called synovial osteo-chondromatosis. In tenosynovial chondromatosis, common sites include the flexor tendons in the fingers, feet, wrist, hands and ankles [1].

## Case report

### Clinical presentation

**Fig. 1:**
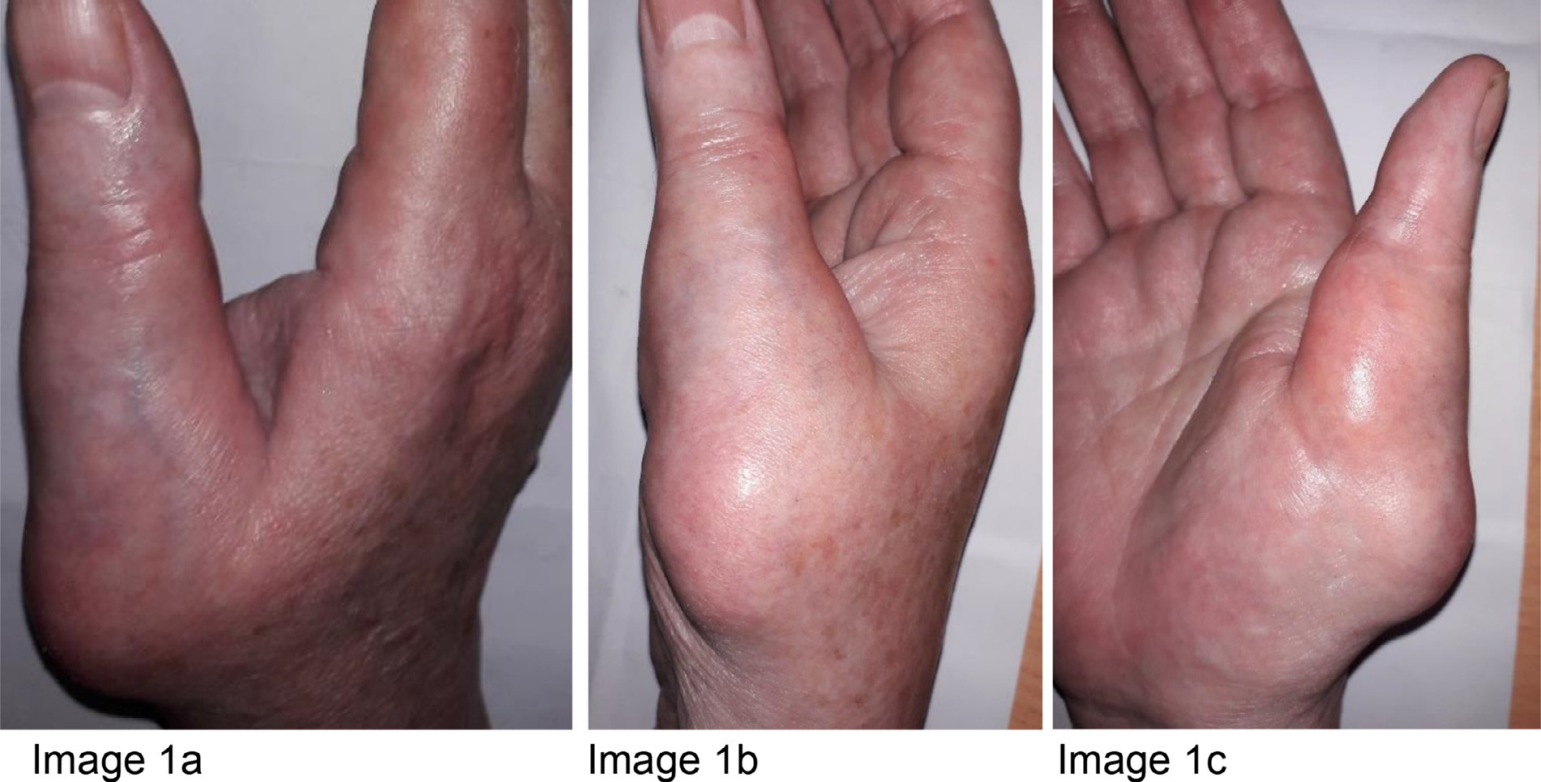
(a-c) Photographs of the patient's right thumb from the dorsal aspect (a), the lateral aspect (b) and the palmar aspect (c). These photographs show the swelling involving the base of the right thumb and thenar eminence.

A 61-year-old female presented with discomfort and increasing size of a lump involving her right thumb and thenar eminence (Figs. 1-c) that had been present for at least 5 years. She had a background of osteoarthritis, in her right thumb of note, hypertension and hypercholesterolaemia. She is an ex-smoker and consumes 20-25 standard drinks of alcohol weekly. Family history was non-contributory.

On examination, there was a circumferential mass extending from the base of the right thumb to the proximal phalanx that was mildly tender. There was no movement at the metacarpophalangeal or interphalangeal joints. Neurovascular supply was intact.

**Fig. 2:**
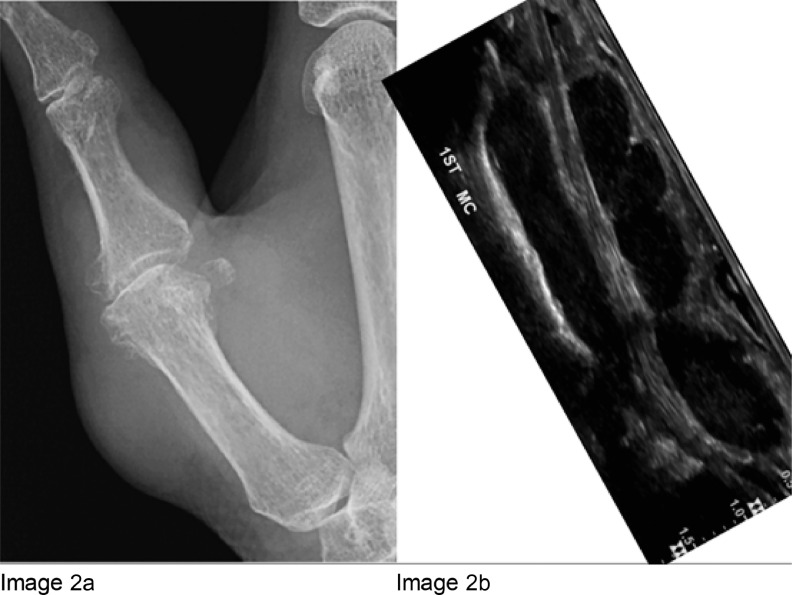
Image 2a: PA radiograph of the right thumb. There is soft tissue swelling over the dorsolateral aspect of the thumb without any underlying bony scalloping or erosion. There are degenerative changes at the metacarpophalangeal articulation of the thumb. Image 2b: Ultrasound of the thumb. The ultrasound displays a multilobulated hypoechoic lesion measuring up to 6cm, surrounding the right flexor pollicis longus (FPL) tendon. The FPL tendon is the hyperintense linear structure.

**Fig. 3:**
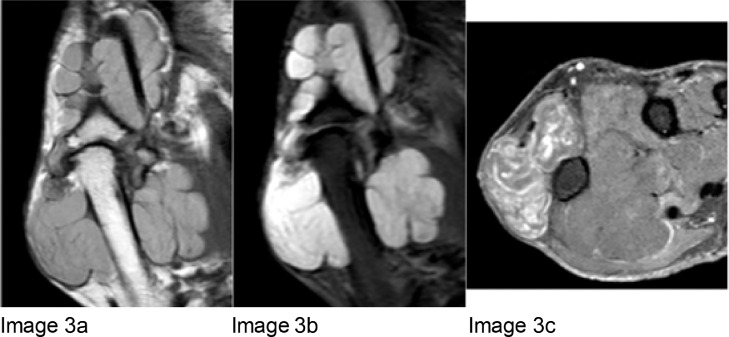
Images 3a-c: Coronal T1 MRI (Image 3a), Coronal T2 MRI (Image 3b), and axial post contrast MRI (Image 3c). The above images display a multilobulated mass encasing the FPL, surrounding the right first proximal phalanx, first metacarpophalangeal joint and first metacarpal, measuring 4.6 × 2.9 × 6.7 cm (width x depth x length). The lesion displays intermediate T1 signal (Image 3a), T2 hyperintense signal (Image 3b) and heterogenous post contrast enhancement (Image 3c).

Initially, the patient had a radiograph of the right hand, which displayed a soft tissue swelling over the dorsolateral aspect of the thumb without any underlying bony scalloping or erosion (Fig. 2). An ultrasound was carried out soon after the X-ray, which displayed a multilobulated hypoechoic lesion measuring up to 6 cm surrounding the right flexor pollicis longus tendon (FPL) (Fig. 3). The imaging differential at this point included giant cell tumour of the tendon sheath, and fibroma of the tendon sheath given that there were no aggressive features such as bony erosion.

**Fig. 4:**
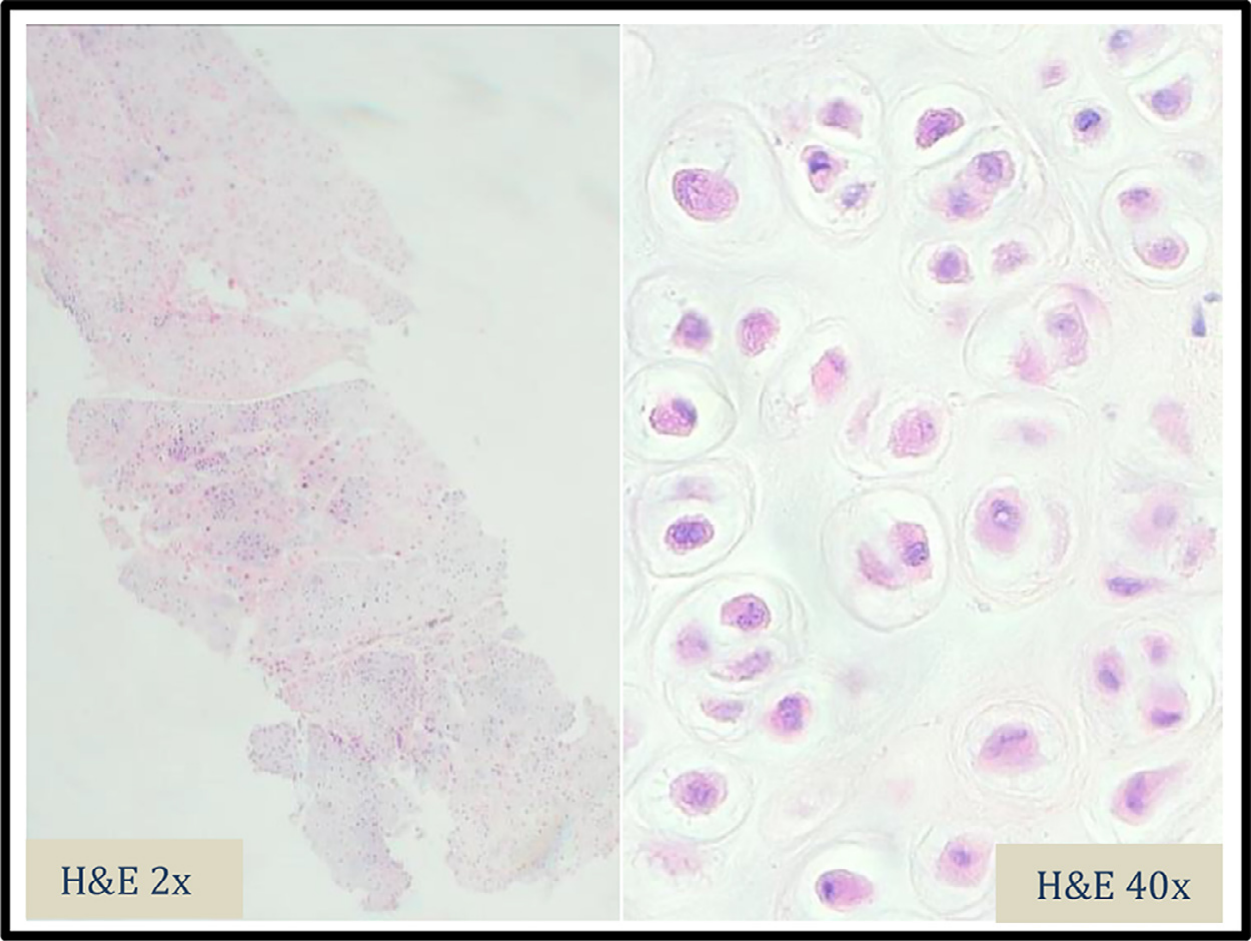
Image 4: H&E (Haemotoxylin and Eosin) stains of the initial biopsy, magnified by 2 and 40 times. These slides show lobules of mature cartilage with mild to moderate cytological atypia. Binucleate lacunae identified. No myxoid area or bony infiltration was noted.

MRI was then performed, which displayed a multilobulated mass encasing the FPL, surrounding the right first proximal phalanx, first metacarpophalangeal joint and first metacarpal (Figs. 4a-c).

### Histologic findings

**Fig. 5:**
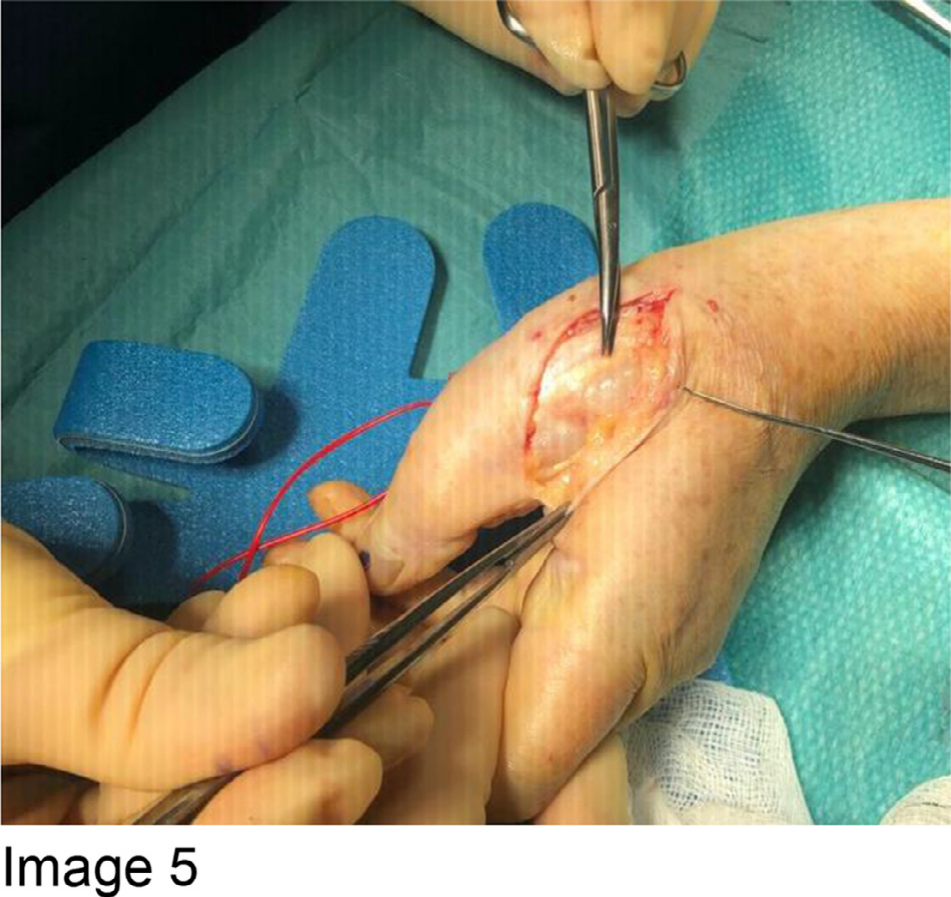
Image 5 : Intraoperative photographs during resection of the tumour (Patient consent was obtained). The photographs show the exposed pale lobulated mass prior to complete removal.

Ultrasound-guided biopsy was performed, which revealed an atypical cartilaginous lesion with mild-to-moderate cytological atypia (Fig. 5). The lesion abutted the bone, without bone infiltration. High-grade malignant features were not identified. The histologic analysis alongside with radiological description, including large size of the lesion, raised the possibility of a low-grade chondrosarcoma. Excision surgery was undertaken.

### Treatment and outcome

**Fig. 6:**
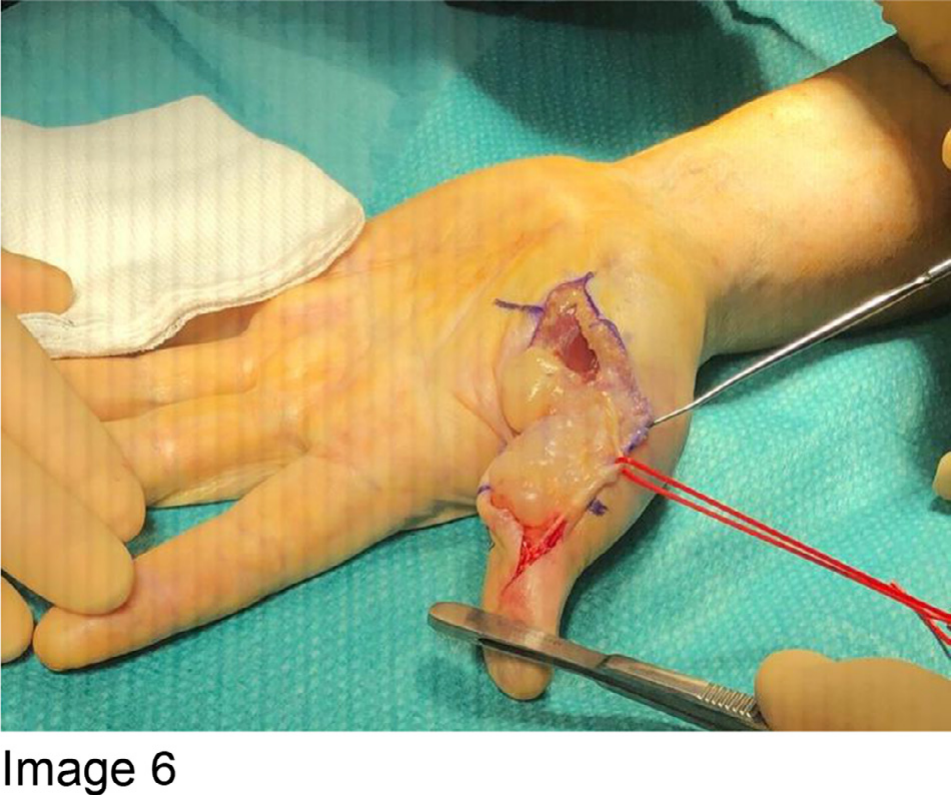
Image 6: The neurovascular bundle is protected, as indicated by the red loop (Figure 6).

**Fig. 7:**
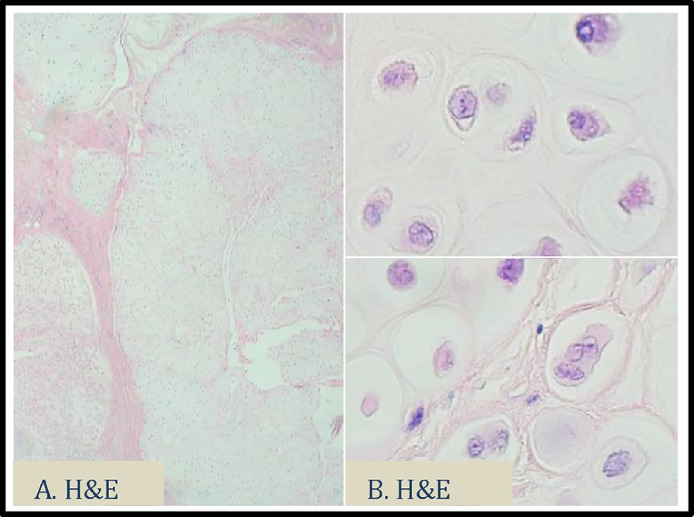
H&E stains of the subsequent excision. These slides show lobules of cartilage in soft tissue (A) and moderate cytological atypia with up to 4 chondrocytes per lacuna (B). No bony infiltration or destruction was noted.

The tumour was resected in its entirety using a dual incision approach (Figs. 5 and 6) centred over the first metacarpal and proximal phalanx. The neurovascular bundle was dissected out and protected throughout (indicated by red vessel loop in Fig. 6). Following tumour resection, the A1 pulley of the thumb flexor tendons was reconstructed using a suture anchor technique (Fig. 7).

The patient was discharged with routine follow-up and surveillance planned. Resection resolved a lot of this lady's pain; however, her osteoarthritis leaves some residual pain and stiffness. There is also some residual weakness of thumb flexion. Overall, she is pleased with her outcome.

Final histopathologic analysis displayed lobules of cartilage in soft tissue. The lesion was well circumscribed and did not display infiltration. Although moderate cytological atypia with up to 4 chondrocytes per lacuna and single chondrocyte necrosis were present, aggressive features such as bony infiltration or destruction were not identified. These histologic findings with multidisciplinary input of the clinical presentation and radiology pattern favoured diagnosis of tenosynovial chondromatosis with cytological atypia.

## Discussion

Milgram, in 1977, described 3 classic stages of synovial chondromatosis: (1) early disease characterized by synovial neoplastic activity, (2) transitional stage with development of loose bodies and (3) late stage with loose bodies but no active synovial disease [3].

In general, in primary synovial chondromatosis, patients are aged between 40 and 60 years and men are affected more commonly than women [1,2]. In the intra-articular form, patient symptoms include pain, swelling and restriction of motion. However, in extra-articular chondromatosis, limitation of range of motion and pain are less common [1,4]. Symptoms will depend on which joint, bursa or tendon sheath is involved. Tenosynovial chondromatosis has a particular predilection for the flexor tendons, tending to spare the extensor tendons [4]. Presentation is usually with painless swelling, slowly increasing over many years. Given the location, trigger-finger or carpel-tunnel symptoms can occur [4].

On radiograph, if the bodies are calcified, they will show a pathognomonic appearance with rounded intra-articular loose bodies of a similar shape and size displaying a chondroid (ring-and-arc) matrix [1,2]. The adjacent joint will be normal. However, in cases of early Milgram stage where there is not yet any mineralization, as in our case, X-rays may be normal or suggest a soft tissue mass. There can be local bone erosion; however, there should be no aggressive bone features or periosteal reaction [4]. Ultrasound can be used to characterize vascularity, relationship to adjacent structures and be used in guiding biopsy.

CT can be used to further characterize the loose bodies, but MRI is best for displaying the relationship to the joint space, bursa or tendon sheath. MRI will generally display a lobulated, homogenous mass with low-to-intermediate signal T1 intensity and T2 hyperintensity [1]. Calcified bodies will be noted as focal areas of signal void. MRI will display the anatomy clearly, and an intimate relationship to the tendon sheath is seen in tenosynovial chondromatosis, as in Figs. 4a and b. There have been similar cases of tenosynovial chondromatosis published in the literature [4], [5], [6], [7], [8], [9], [10]. Our case is unusual because of the lack of calcification, which is seen in 90% [10].

Macroscopic features of this soft tissue mass are multiple small (0.1-1.0 cm) cartilaginous lobules that coalesce into large conglomerates [11,12]. Histopathologic analysis displays lobules of hyaline-myxoid cartilage, chondrocytes arranged in small clusters and cytologic atypia with some increase cellularity [1,^11^,12]. The histologic differential diagnosis of synovial chondromatosis includes: osteocartilaginous loose bodies, soft tissue chondroma, juxta-articular chondroma and synovial chondrosarcoma, which may arise within synovial chondromatosis or de novo. The histopathologic diagnosis of cartilaginous tumours poses a challenge, as they can exist along a spectrum of aggressiveness. Histologic assessment can therefore be difficult with varying degrees of hypercellularity, necrosis, multinucleation, nuclear crowding, nuclear enlargement and mitotic figures. It is worth noting that benign cartilaginous tumours may display atypical features and also sometimes undergo frank malignant transformation [13]. This case is unusual in that synovial chondromatosis is present as large (6 cm) lesion in small metacarpal joint. In histopathology, standard of care is not to report bone tumours without radiological correlation [14]. Therefore, exclusion of low-grade chondrosarcoma solely based on histopathologic features is not recommended and clinico-radiological correlation is always advised [15,16].

Given that each line of approach (clinical, radiological and histologic) has certain limitations, it is important to correlate clinical history, imaging appearances and histologic appearance with multidisciplinary team input. Clinical features that are concerning for a more aggressive process include pain and rapid growth. Radiological features of concern include deep cortical scalloping, cortical destruction or a soft tissue mass.

Treatment is surgical synovectomy with removal of chondral fragments. Recurrence is higher in extra-articular (up to 88% [10]) than intra-articular chondromatosis and therefore surgery and surveillance should be aggressive [2]. Malignant transformation can occur in up to 5% [1].

### Learning points

• In cartilaginous tumours, it can be difficult to outrule a low-grade chondrosarcoma by histology alone; correlation of clinical, imaging and histologic features is important.
• Primary synovial chondromatosis can be intra-articular or extra-articular (tenosynovial or bursal chondromatosis).
• Tenosynovial chondromatosis typically affects flexor tendons of the hands or feet.
• Tenosynovial chondromatosis can be present for many years, and be relatively asymptomatic, prior to diagnosis.
• Factors concerning for low-grade chondrosarcoma: hypercellularity, cytological atypia, myxoid change and necrosis.

## Patient Consent

We confirm that written, informed consent for publication was obtained from the patient.
